# Isolated primary amyloidosis of the inferior rectus muscle mimicking Graves’ orbitopathy

**DOI:** 10.1590/S1679-45082016RC3744

**Published:** 2016

**Authors:** Mário Luiz Ribeiro Monteiro, Allan Christian Pieroni Gonçalves, Alanna Mara Pinheiro Sobreira Bezerra

**Affiliations:** 1 Faculdade de Medicina, Universidade de São Paulo, São Paulo, SP, Brazil.; 2 Hospital Israelita Albert Einstein, São Paulo, SP, Brazil.

**Keywords:** Graves disease/diagnosis, Orbital diseases, Oculomotor muscles/physiopathology, Amyloidosis, Tomography, x-ray computed, Case reports

## Abstract

The diagnosis of Graves’ orbitopathy is usually straightforward. However, orbital diseases that mimick some clinical signs of Graves’ orbitopathy may cause diagnostic confusion, particularly when associated to some form of thyroid dysfunction. This report describes the rare occurrence of localized inferior rectus muscle amyloidosis in a patient with autoimmune hypothyroidism, who was misdiagnosed as Graves’ orbitopathy. A 48-year-old man complained of painless progressive proptosis on the left side and intermittent vertical diplopia for 6 months. The diagnosis of Graves’ orbitopathy was entertained after magnetic resonance imaging revealing a markedly enlarged, tendon-sparing inferior rectus enlargement on the left side, and an autoimmune hypothyroidism was disclosed on systemic medical workup. After no clinical improvement with treatment, the patient was referred to an ophthalmologist and further investigation was performed. The presence of calcification in the inferior rectus muscle on computed tomography, associated with the clinical findings led to a diagnostic biopsy, which revealed amyloid deposition. This report emphasizes that a careful evaluation of atypical forms of Graves’ orbitopathy may be crucial and should include, yet with rare occurrence, amyloidosis in its differential diagnosis.

## RESUMO

O diagnóstico de orbitopatia de Graves usualmente é fácil de ser estabelecido. No entanto, doenças da órbita que simulam alguns sinais clínicos da orbitopatia de Graves podem levar à confusão diagnóstica, particularmente quando associada à alguma forma de disfunção tireoidiana. Relatamos a ocorrência rara de amiloidose localizada no músculo reto inferior em paciente com hipotireoidismo autoimune, que recebeu inicialmente o diagnóstico errôneo de orbitopatia de Graves. Paciente masculino, 48 anos, com queixa de proptose progressiva e indolor do lado esquerdo e diplopia vertical intermitente há 6 meses. O diagnóstico de orbitopatia de Graves foi considerado após a realização de ressonância magnética, que revelou aumento importante do músculo reto inferior esquerdo, sem acometimento do tendão, e uma propedêutica sistêmica detectou hipotireoidismo autoimune. Como não houve melhora com o tratamento clínico, o paciente foi encaminhado a um oftalmologista, que realizou nova investigação. A presença de calcificação no músculo reto inferior na tomografia computadorizada, associada aos achados clínicos, levou a uma biópsia da lesão, que demonstrou a deposição de material amiloide. Este relato enfatiza como uma avaliação minuciosa das formas atípicas de orbitopatia de Graves é essencial e deve incluir a ocorrência, embora rara, de amiloidose no diagnóstico diferencial da orbitopatia de Graves.

## INTRODUCTION

Graves’ orbitopathy (GO) refers to a combination of adnexal and orbital findings caused by an immune-mediated inflammatory process, which induces expansion of the extraocular muscles and orbital fat. Its pathophysiology is closely related to that of Graves’ disease.^(1)^ Typical findings in GO include proptosis, eyelid retraction, periorbital edema, chemosis and restrictive ophthalmoplegia that may precede, coincide with, or follow systemic signs of thyroid disease. Upgaze is typically limited initially, since the inferior rectus muscle is the most frequently involved extraocular muscle in GO.^(2)^

Based on the clinical signs and laboratory testing for thyroid function, the diagnosis of GO is usually straightforward. However, a few orbital diseases can mimick the clinical findings of GO leading to great diagnostic confusion, particularly when the patient has been previously diagnosed as suffering from thyroid disorders.^(3)^

This report describes a patient with autoimmune hypothyroidism, who later presented unilateral proptosis from an enlarged inferior rectus muscle observed on magnetic resonance imaging. The patient was initially misdiagnosed and treated by other physicians as GO. Considering an atypical limitation of depression of the eye and presence of calcification on imaging studies, an alternative diagnosis was entertained and the patient was subsequently found to have primary isolated amyloidosis of the orbit.

## CASE REPORT

**Figure 1:**
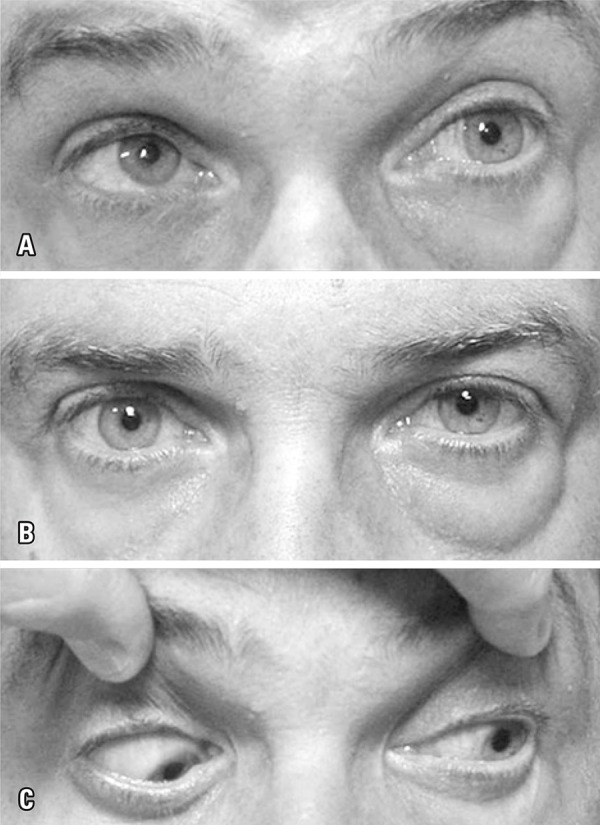
Photographs at presentation showing left lower eyelid bag swelling, left proptosis and extraocular motility *deficits*. (A) Left upgaze; (B) Primary gaze; (C) Left downgaze

**Figure 2:**
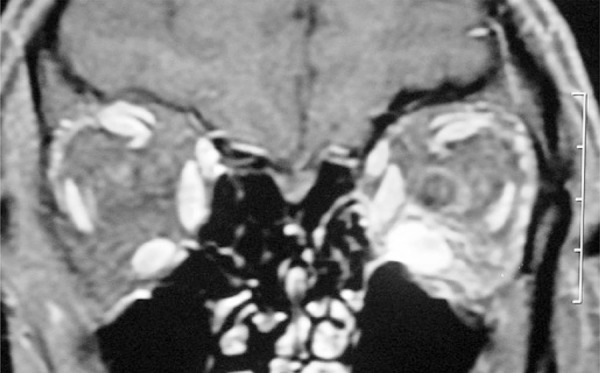
Coronal T1-weighted gadolinium-enhanced magnetic resonance imaging of the orbits showing marked hyperintense enlarged inferior rectus muscle on the left

A 48-year-old man complained of painless progressively enlarging lower eyelid bag swelling, proptosis on the left side and vertical diplopia for 6 months (Figure 1). A systemic medical workup disclosed autoimmune hypothyroidism and no other remarkable findings. Magnetic resonance imaging demonstrated a significantly enlarged, tendon-sparing inferior rectus muscle on the left side (Figure 2). Based on such findings, the diagnosis of GO was entertained. Hypothyroidism was controlled with proper medication. High-dose oral prednisone was prescribed for 2 months, but no clinical improvement occurred and the patient was referred to us for specialized orbital consultation.

**Figure 3:**
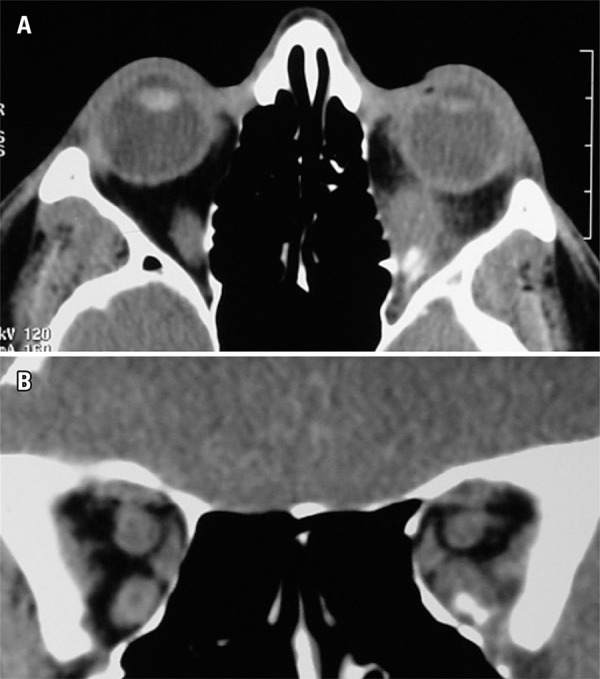
Computed tomography scans of the orbits show enlarged left inferior rectus muscle with an infiltrated and calcified lesion. (A) Axial view; (B) Coronal view

On examination, visual acuity was normal in both eyes. There was a 4-mm left proptosis, but no lid retraction on either side. Extraocular motility revealed both marked upgaze (-3 superior rectus) and downgaze (-2 inferior rectus) *deficits* (Figure 1). Extraocular motility of the right eye was normal. Because of inferior rectus depression *deficit* and absence of lid retraction, the diagnosis of GO was questioned and an orbital computed tomography scan was obtained. While the diagnosis of GO was still considered by the radiologist, the presence of discrete but well documented calcification in the inferior rectus muscle (Figure 3) suggested an alternative diagnosis and a muscle biopsy was performed.

**Figure 4:**
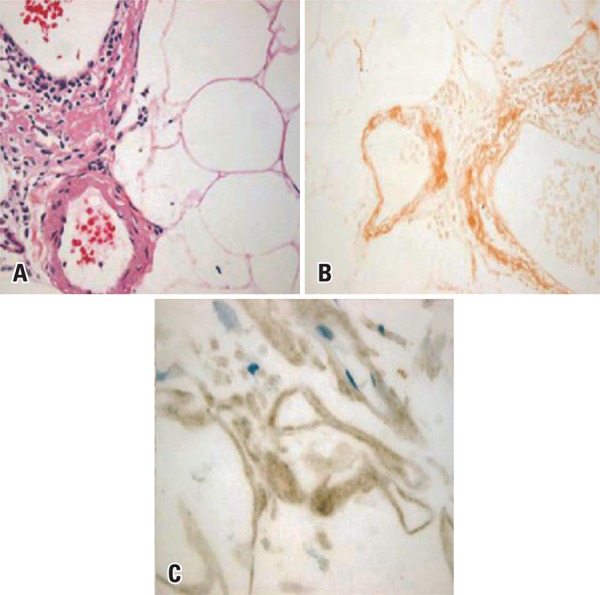
Histopathological study of the inferior rectus muscle of a patient with primary localized amyloidosis. (A) Extracellular amorphous hyaline eosinophilic material in fibroadipose tissue (hematoxylin-eosin, 40X magnification); (B) Congo red staining disclosing extracellular material (40X magnification); (C) Immunohistochemistry study with positive amyloid A protein staining (40X magnification)

The histopathological examination of tissue biopsies revealed extracellular amorphous and hyaline eosinophilic material on hematoxylin-eosin, and orange-red staining with Congo red showed a green birefringence (Figures 4A and 4B). Immunohistochemistry showed positive amyloid A protein staining (Figure 4C). Based on such findings, the histopathological diagnosis was defined as amyloid deposits. A thorough systemic investigation was completely unrevealing and the diagnosis of primary localized orbital amyloidosis was made. No amyloid deposition was found elsewhere in the body.

## DISCUSSION

Graves’ orbitopathy shows great variability of presenting symptoms and signs. Graves’ orbitopathy is usually bilateral, and the most common cause of bilateral or even unilateral exophthalmos among adults.^(4)^ Although eyelid retraction is its major associated sign, the diagnosis of GO may also be made based on proptosis and strabismus.^(2)^ Graves’ disease presenting as hyperthyroidism is the underlying diagnosis in the majority (80%) of individuals with GO, but patients may present with primary autoimmune hypothyroidism or with no past or present history of thyroid dysfunction.^(2)^

Despite the fact that GO has a wide spectrum of clinical presentations, when atypical features of GO diagnosis outweigh the typical expected findings, clinicians ought to reconsider their first presumptive diagnosis. As in our report, the atypical ocular motility disturbance, lack of eyelid retraction and unilateral presentation, should alert the physician to pursue further diagnostic investigation. In fact, when the inferior rectus muscle in involved in GO, extraocular motility is usually manifested by restrictive muscle dysfunction. Therefore, patients do present limitation of upgaze, as demonstrated by our patient, but do not usually display inferior *rectus* function *deficit*. Thus, the presence of significant *deficit* of eye depression in our case was an important clinical finding to question the diagnosis of GO (Figure 1, bottom).

Imaging studies play an important role in the differential diagnosis of GO.^(5)^ In our case, computed tomography showed enlargement of the inferior rectus muscle with a marked infiltrative calcified lesion driving the diagnosis for new possibilities. However, magnetic resonance imaging is unable to detect calcified lesions and, as a single study, could have reinforced the clinical misdiagnosis.

There are numerous underlying causes for enlarged extraocular muscles: GO; orbital inflammation, such as sarcoidosis or nonspecific (myositis); infections, as in Lyme disease, cysticercosis or trichinosis; vascular conditions, such as carotid cavernous fistulas or arteriovenous malformation; myopathies; acromegaly; infiltrative disorders; and neoplastic disease.^(6,7)^ Calcified orbital lesions are described in metastatic tumors of the orbit, meningioma, teratoma, neurofibroma, sclerosing hemangioma, chondrosarcoma and amyloidosis.^(8)^

The clinical and imaging features of this case narrowed the possibilities to a neoplastic disease or a deposition disorder. Neoplastic involvement of the extraocular muscles may be caused by local infiltration by adjacent tumors, including primary orbital tumors and secondary neoplasms from periorbital sites, or by metastases from distant sites.^(9)^ Amyloidosis is a deposition disorder that may be localized or systemic, primary or secondary to chronic inflammatory diseases. Orbital involvement is more common in the primary form of amyloidosis.^(10)^ In both types of disease, after a negative systemic workup, tissue biopsy of the muscle affected is required for certain diagnoses.

## CONCLUSION

In conclusion, a rare case of localized orbital amyloidosis to the extraocular muscle, misdiagnosed as Graves’ orbitopathy, was reported. The authors stressed the importance of a careful diagnosis of some atypical Graves’ orbitopathies, including amyloidosis, despite its rare occurrence, in their differential diagnosis.
